# Bone Health Optimization in Adult Spinal Deformity Patients: A Narrative Review

**DOI:** 10.3390/jcm13164891

**Published:** 2024-08-19

**Authors:** Yousef A. Al-Najjar, Danyal A. Quraishi, Neerav Kumar, Ibrahim Hussain

**Affiliations:** Department of Neurological Surgery, Och Spine at New York Presbyterian at the Weill Cornell Medical Center, 525 East 68th Street, New York, NY 10065, USA; yaa4004@qatar-med.cornell.edu (Y.A.A.-N.); danyalquraishi@gmail.com (D.A.Q.); nek4003@med.cornell.edu (N.K.)

**Keywords:** osteoporosis, scoliosis, kyphosis, compression fracture, spinal fracture, diphosphonates, denosumab, teriparatide

## Abstract

Osteoporosis and low bone mineral density (BMD) pose significant challenges in adult spinal deformity surgery, increasing the risks of complications such as vertebral compression fractures, hardware failure, proximal junctional kyphosis/failure, and pseudoarthrosis. This narrative review examines the current evidence on bone health optimization strategies for spinal deformity patients. Preoperative screening and medical optimization are crucial, with vitamin D supplementation showing particular benefit. Among the pharmacologic agents, bisphosphonates demonstrate efficacy in improving fusion rates and reducing hardware-related complications, though the effects may be delayed. Teriparatide, a parathyroid hormone analog, shows promise in accelerating fusion and enhancing pedicle screw fixation. Newer anabolic agents like abaloparatide and romosozumab require further study but show potential. Romosozumab, in particular, has demonstrated significant improvements in lumbar spine BMD over a shorter duration compared to other treatments. Surgical techniques like cement augmentation and the use of larger interbody cages can mitigate the risks in osteoporotic patients. Overall, a multifaceted approach incorporating medical optimization, appropriate pharmacologic treatment, and tailored surgical techniques is recommended to improve outcomes in adult spinal deformity patients with compromised bone quality. Future research should focus on optimizing the treatment protocols, assessing the long-term outcomes of newer agents in the spine surgery population, and developing cost-effective strategies to improve access to these promising therapies.

## 1. Introduction

Spinal deformities are one of the most common medical disorders with significant impact on the patients’ quality of life. With a significant burden on healthcare costs, it is estimated that 27.5 million elderly patients suffer from some form of spinal deformity [1]. Spinal deformity is a heterogenous spectrum of disorders defined as the malalignment or malrotation of the spine in the axial, coronal, and/or sagittal plane. Specific subtypes of spinal deformities include scoliosis, kyphosis, sagittal malalignment, spondylolisthesis, axial plane deformity, and rotary subluxation [2,3]. The prevalence of different types of spinal deformities differs depending on various factors such as age, genetics, comorbidities, and lifestyle. Scoliosis, for example, is prevalent in childhood and adolescent populations, with the most common subtype being idiopathic scoliosis accounting for 80% of spinal deformities in pediatric patients [4]. The progression of adolescent spinal deformities into adulthood, degenerative changes associated with age, and iatrogenic deformities post-surgery impose a huge risk for the development of de novo spinal deformities in adult and elderly populations [1,2,3]. Among the most cited factor in the development of spinal deformity or the progression of deformity post-surgery is low bone mineral density-related conditions. Suboptimal management of this can lead to poorer outcomes and therefore requires close attention in the preoperative optimization of patients planned for reconstruction surgery [5,6,7].

This narrative review aims to explore how different pharmacological agents affect fusion rates and hardware-related complications in adult spinal deformity patients with osteoporosis, as well as the most effective preoperative bone health optimization strategies for improving surgical outcomes in adult spinal deformity patients with low bone mineral density.

## 2. Osteoporosis and Osteopenia

While there are several factors implicated in the pathogenesis of spinal deformities, smoking, weight, core muscle strengthening, as well as general lifestyle, career and activity choices are among those that can be modified to improve quality of life and outcomes from surgery [8,9,10]. In terms of comorbid modifiable medical conditions, osteoporosis is a disorder of the bone whereby decreased bone mass is coupled with increased risk of fragility fractures [11,12]. A significant public health concern, consistently cited as the most common bone disease in humans, osteoporosis was shown in a meta-analysis to be prevalent in 18.3% of the global population and 23.1% of women [13,14]. On a broad basis, osteoporosis is one of many bone demineralizing diseases that falls under the umbrella term osteopenia [15]. Now recognized as a variable decrease in the bone mineral density but not low enough for the diagnosis of osteoporosis, osteopenia is more common in men and postmenopausal women than osteoporosis, with more than half of postmenopausal women in the US developing osteopenia [12,16,17,18]. Among the lifestyle choices that affect the development of osteoporosis and osteopenia and the maintenance of bone health are included nutrition (specifically calcium and vitamin D), smoking, exercise, alcohol use, body mass index (BMI) (low BMI or body weight is usually associated with an increased risk of osteopenia and osteoporosis), occupation, recreational/sport activities, and caffeine consumption [11,12,15,16,17,19,20]. Other risk factors include inflammatory conditions, recurrent infections, HIV, malabsorptive diseases, diabetes mellitus, and certain medications [11,12,15,16,17]. Risk factors such as age, ethnicity and sex are also heavily involved in the pathogenesis of primary osteoporosis while the other risk factors, most of which are modifiable, are involved in the pathogenesis of secondary osteoporosis [11,21]

## 3. Risks and Complications in Osteoporotic Patients

**Figure 1:**
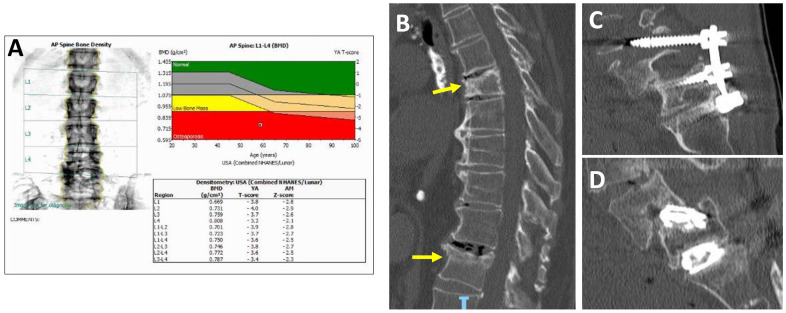
Spinal pathology due to osteoporosis. (**A**) Bone densitometry scan (DEXA) demonstrating a postmenopausal woman with osteoporosis of the lumbar spine, defined as a T-score (comparison with healthy adults) less −2.5 and a Z-score (comparison with similar peer group) less than −1.5, Green is normal bone density, yellow is low bone mass, and red is osteoporosis. (**B**) Acute vertebral compression fractures (yellow arrows). (**C**) Hardware failure with pedicle screw loosening, osteolysis, and migration. (**D**) Interbody cage subsidence, nonunion, and resultant spondylolisthesis.

Osteoporosis is an essential parameter for surgeons, as many patients undergoing spinal surgery have compromised bone quality [22,23] and adverse outcomes are directly proportional to the complexity of the deformity [24,25]. The incidence of osteoporosis in patients undergoing spine surgery who are older than 50 years is reported to be 14.5% of men and 51.3% of women [26,27]. This interrelation warrants attention, as adult spinal deformity in the United States has increased 3.4-fold in the past decade [28,29]. Both osteoporosis and osteopenia are associated with vertebral fractures after instrumentation, hardware failure, proximal junction kyphosis, and pseudoarthrosis [22,23,27,30,31]. and carry an increased risk of revision surgery and surgical complications within two years of the procedure [32] (Figure 1). Gupta et al. found that of 399 adult spinal deformity patients, 131 of whom had osteoporosis, 40% needed revision surgery, which is 1.45 times more likely than those without osteoporosis [32]. With degenerative scoliosis affecting 69% of the elderly population, it is apparent that bone mineral density, advanced age, and spinal deformity are associated characteristics that warrant careful surgical planning before surgical intervention is conducted [33,34]. Below are the commonly cited complications presented in osteoporotic and osteopenic patients undergoing spinal surgery.

### 3.1. Vertebral Compression Fractures

Vertebral compression fractures (VCF) compromise the anterior column of the spine, placing excessive strain on the anterior portion of the vertebra and the anterior longitudinal ligament [35]. The most common fractures due to osteoporosis are within the vertebrae, as osteoporosis in the aging spine significantly increases the risk of vertebral compression fractures and proper fixation [27,36]. Although widely under-reported, as many VCFs go undetected, VCFs comprise approximately 700,000 of the total 1.5 million annual osteoporotic fractures in the USA [31,37,38].

**Figure 2:**
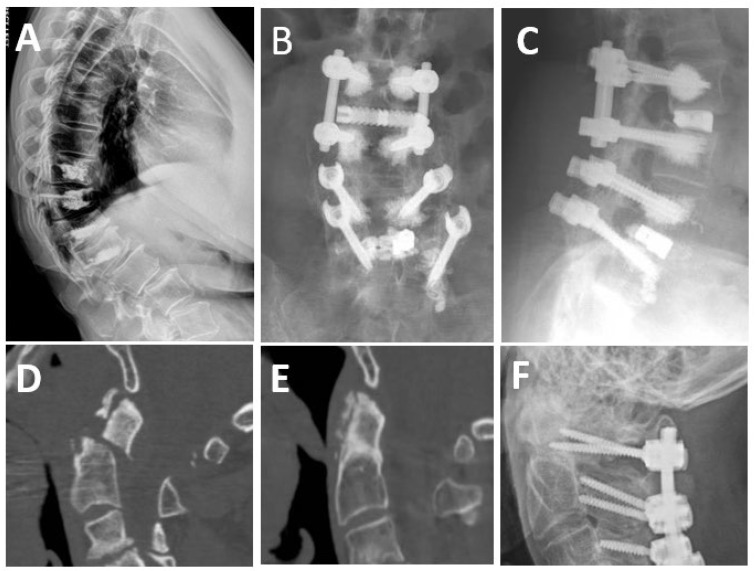
Surgical treatment options for patients with osteoporosis. (**A**) Four-level kyphoplasty in an elderly woman with multiple thoracic vertebral compression fractures and thoracic hyperkyphosis. (**B**,**C**) AP and lateral X-rays from a hardware revision and fusion extension in a patient with osteoporosis and adjacent segment disease (ASD) at L2/3 following solid arthrodesis from previous L3–5 fusion. Note the new screws at L2 and L3 with cement augmentation through fenestrated screws. Larger interbody cages, such as those that can be inserted from an anterolateral approach, rest on the apophyseal ring of the vertebrae and thus provide stronger anterior column support and higher fusion rates. (**D**) Preoperative sagittal CT scan of a patient with advanced osteoporosis and a type II traumatic odontoid fracture. (**E**,**F**) Postoperative sagittal CT and lateral X-rays of the same patient 6 months following surgical stabilization and teriparatide treatment, demonstrating reduction and healing of the fracture.

VCF presentation is highly variable, ranging from severe pain causing hospital admission to minor to no symptoms, and is incidentally found on imaging [31,35]. Symptomatic presentation tends to consist of severe focal back pain, functional disability, and progressive kyphosis of the thoracic spine that ultimately results in decreased appetite, poor nutrition, and impaired pulmonary function [35,38,39]. Neurologic symptoms may arise due to spinal cord or cauda equina compression, including increased kyphosis, hyporeflexia, hyperreflexia, sensory loss, urinary retention, and sphincter dysfunction [35,38,39]. Irrespective of clinical presentation, VCFs are key clinical markers for skeletal fragility, as one VCF increases the risk of a future, potentially more harmful, fracture by 5-fold [31,35,36,39]. With respect to spinal deformities, VCFs can cause the progression of pre-existing curvature abnormalities [24,38]. The loss of height that results from a compression fracture may lead to the worsening of kyphotic deformity of the spine. This is especially true for multiple compression fractures where significant height loss is a concern. Management can range from conservative pharmacologic analgesia, bracing, percutaneous vertebroplasty or kyphoplasty, and corrective surgeries with instrumentation and interbody grafts [35,38,40] (Figure 2A).

### 3.2. Hardware Failure

Osteoporotic bone is known to exhibit decreased pullout strength and insertional torque [41,42]. This characteristic makes patients more vulnerable to screw toggling, loosening, and eventual pullout [25,32,41,43]. These complications are compounded with multilevel adult spinal deformity constructs, as even stronger forces are placed on the patient’s bone to maintain substantial correction and withstand the forces of daily living activities [32]. Bone mineral density (BMD) has been cited as one of the most important factors to consider when assessing the risk of screw pullout and bone-screw interface failure [22,30]. In a recent systematic review and meta-analysis by Ogiri et al., consisting of 133,000 patients, 12% of which had osteoporosis, those with compromised bone quality were 2.59 times more likely to have screw loosening and 1.65 times more likely to need revision surgery compared with those with standard bone quality [22]. Similarly, Rometsch et al. found that in their systematic literature review of the incidence of screw loosening in osteoporotic spines, pedicle screw loosening rates were twice as high as in patients with regular bone density when the screws were placed in a nonaugmented fashion [42].

To prevent hardware malfunctions, some papers have cited that a spine with poor bone mineral density may require multiple fixation points, including adjunct fixation and augmentation of pedicle screws, and the use of larger interbody cages [24,42] (Figure 2B,C). Additionally, large-diameter, long pedicle screws with or without fenestrations for cement augmentation can structurally fill more of the pedicle, maintaining the integrity of a solid construct in osteoporotic patients [32,41]. Cement augmentation in vertebroplasty and kyphoplasty provides significant pain relief and vertebral height restoration with minimal invasiveness, but it carries risks of bone cement implantation syndrome (BCIS), which can result in severe complications such as hypoxia, hypotension, and cardiovascular collapse, potentially leading to fatal outcomes [44,45]. Several other risks exist like cement leakage, adjacent fractures, and other surgical complications; however, risk mitigation can be performed through medical intervention with agents such as bisphosphonates, calcitonin, abaloparatide, and teriparatide [32,44,45].

### 3.3. Proximal Junctional Kyphosis and Failure

Proximal junctional kyphosis (PJK) is a radiographic phenomenon that demonstrates kyphosis in which the proximal junction angle (the sagittal Cobb angle between the inferior endplate of the upper instrumented vertebra and the superior endplate of the vertebra two levels above) becomes more than 10 degrees [23,46,47,48]. PJK is often diagnosed when spinal deformity patients return for follow-up with unremitting pain at the top of the construct [49]. Patients with either osteoporosis or osteopenia undergoing lumbar fusions have an elevated risk for PJ [30,41,43]. This risk is compounded within patients with adult spinal deformity [29,47,48]. Proximal junctional failure arises upon symptom onset, vertebral collapse, or instrumentation failure and carries a substantial risk of sudden paralysis [46,48]. Retrospective studies have shown that pre-existing low BMD is an independent risk factor for proximal junctional failure (PJF), with a 2% incidence rate [46].

In a recent systematic review and meta-analysis by Ogiri et al., consisting of 133,000 patients, 12% of whom had osteoporosis, the risks of PJK/PJF were 1.89 higher in those with poor bone quality than in those with healthy bone [22]. Similar associations were found in the lens of adult spinal surgery, as Kuo et al. found that in their retrospective chart review of 116 patients who had received ASD surgery, a vertebral bone quality score of 2.85 or higher was independently associated with PJK/PJF occurrence with a 94.3% predictive accuracy [29]. Another study of 113 patients surgically treated for spinal deformity were grouped as having either mildly low to normal BMD (T-score > −1.5) or significantly low BMD (T-score < −1.5) and found that the incidence of PJF was significantly higher in the patient group with significantly low BMD (33% occurrence) when compared with patients with normal BMD (8% occurrence) [46].

To prevent PJK, techniques such as ligament augmentation, vertebroplasty, transverse process hooks, flexible rods, sublaminar tape, and multilevel stabilization screws have been used [48,50]. In their systematic review of studies assessing PJK prevention in ASD surgeries, Doodkorte et al. found that the laminar or sublaminar use of polymeric cable systems showed particularly significant efficacy in PJK prevention in the osteopenic ASD patient population [50]. This corroborates previous studies’ findings indicating that resistance to failure in laminar hook and sublaminar wire fixation was not correlated to overall bone mineral density, which benefits a vulnerable BMD population [51]. Additionally, laminar bone mineral density is relatively higher than other places of fixation, such as the pedicles and transverse processes, which further supports its use in osteopenic ASD patients [52].

## 4. Osteoporosis Treatment and Optimization for Spine Surgery

The appropriate management of the risk factors for osteoporosis and osteopenia, and by extension, spinal deformities, can dramatically reduce the risk of fractures and surgical intervention [11,12,15,16,17,18,32,53]. Both pharmacological and nonpharmacological interventions can also improve pre- and post-surgical bone health optimization thereby minimizing the previously mentioned surgical complications [32]. There are many modalities in which osteoporosis can be treated and they can be classified as nonpharmacological and pharmacological interventions. Nonpharmacological interventions include supplements (particularly calcium and vitamin D), exercise (the nature of which involves biomechanical stress to promote bone remodeling), reduction/optimization of modifiable risk factors (occupational hazards, recreational/sport activities, alcohol consumption, smoking cessation, caffeine consumption) and the appropriate management of medical conditions (diabetes mellitus, recurring infections, inflammatory conditions, HIV, malabsorptive diseases, etc.) [11,12,15,16,19,20,21,54,55]. On the other hand, pharmacological interventions include the use of antiresorptive agents (bisphosphonates, denosumab), parathyroid hormone analogues (teriparatide, abaloparatide), hormonal therapies (estrogen agonists/antagonists, estrogen-progestin therapy, testosterone therapy, calcitonin), and novel therapies and drugs [21,56,57] (Table 1).

### 4.1. Vitamin and Mineral Supplementation

Some of the literature has investigated the use of nonpharmacologic interventions for osteoporosis in improving outcomes following spine surgery. In a study in rats, Cho et al. found that dietary calcium improved the volume and overall mechanical strength of lumbar fusions [58]. However, there are no clinical studies in humans to corroborate these results and support calcium supplementation in spinal surgery. There is more evidence that indicates the use of vitamin D supplementation in spinal surgery. In vivo, Metzger et al. found that rats given higher doses of vitamin D were correlated with higher fusion rates, biomechanical stiffness, and bone density following posterolateral fusion [59]. Several studies in human patients who underwent spinal fusion have shown that vitamin D deficiency, compared to normal or elevated vitamin D levels, is correlated with worse post-operative scores for disability, pain, and quality of life [60,61]. Additionally, vitamin D supplementation improves or even resolves chronic back pain, particularly in patients with failed back surgery [62,63,64].

In a recent randomized controlled trial, Hu et al. found that patients given vitamin D supplements exhibited shorter time to fusion, improved spine function, and decreased pain scores following spinal fusion surgery [65]. A second randomized control study by Krasowska et al. found that the decreased pain experienced by patients with vitamin D supplementation was correlated with lower levels of serum markers for systemic inflammation [66]. Based on this review, substantial evidence supports the use of vitamin D supplements to improve outcomes in patients undergoing spinal fusion surgery. However, there is a clear deficit in and a dire need to increase the number of studies analyzing calcium supplementation in these patients.

### 4.2. Antiresorptive Agents

#### Bisphosphonates

Bisphosphonates are a group of medications that inhibit osteoclast function, thus allowing osteoblasts to more efficiently build bone mass. These include zoledronic acid, ibandronate, alendronate, and risedronate. In a randomized controlled trial, Nagahama et al. found that patients given alendronate following posterior lumbar interbody fusion had a significantly higher incidence of solid fusions, and a lower incidence of cage subsidence and subsequent vertebral fractures [67]. Notably, alendronate was associated with a decrease in bone resorption and formation, suggesting impaired healing of spinal fusion, but the authors argued that the mechanical benefits of alendronate outweighed its deficits in healing [67]. Similarly, a retrospective analysis by Tu et al. found that patients administered with zoledronic acid before lumbar interbody fusion had decreased incidence of vertebral compression fracture, pedicle screw loosening, and cage subsidence, which corroborated with results from a clinical trial [68,69]. As an initial characterization of the temporal nature of the benefits of bisphosphonates, a comparative study reported that fusion rates at 6 months post-surgery were lower in patients that took bisphosphonates but were substantially higher for those patients after 2 years [70].

Several systematic reviews and meta-analyses have attempted to synthesize the results of bisphosphonate use across randomized controlled trials, prospective studies, and retrospective cohort analyses. Meta-analyses by Govindarajan et al. and Liu et al. found that the use of bisphosphonates following spinal fusion increased the odds of successful fusion, decreased the likelihood of postoperative vertebral compression fracture, and significantly reduced the scores for disability and pain [71,72]. Liu et al. and Mei et al. found that patients administered bisphosphonates were significantly less likely to exhibit pedicle screw loosening and cage subsidence, like the findings in a review by Buerba et al. analyzing bisphosphonate use after thoracolumbar spinal fusion [72,73,74]. However, Liu et al. saw no benefits of bisphosphonates in reducing the likelihood of implant fixation failure [72].

Some systematic reviews and meta-analyses in the current literature, however, report conflicting results. For example, meta-analyses by Buerba et al., Cheng et al. and Mei et al. found that bisphosphonate therapy does not improve fusion rate following spinal fusion surgery [73,74,75]. Many reviews also document that bisphosphonate use does not change the rate of screw loosening or improve disability scores [71,73,74,75,76]. However, the specific meta-analyses for these parameters in these reviews are relatively underpowered, excluding papers that are present in other recent analyses and, thus, weakening the reliability of these results.

In general, the current literature surrounding bisphosphonate use in spinal fusion surgery is positive. Individual and pooled analyses both show that bisphosphonates increase the mechanical strength of the fusion, which minimizes post-operative complications and improves patient outcomes.

### 4.3. Anabolic Agents

#### 4.3.1. Denosumab

Denosumab is a human monoclonal antibody that inhibits the receptor activator of nuclear factor kappa-Β ligand (RANKL), resulting in a reduction in osteoclast development. Some studies have analyzed the combination of denosumab and teriparatide in patients undergoing spinal fusion [75,77]. The pooled analyses in Cheng et al. show that patients with both teriparatide and denosumab therapy experienced higher fusion rates compared to placebo controls [75]. In a randomized controlled trial examining patients who underwent posterior lumbar interbody fusion, Ide et al. found that patients treated with a combination of teriparatide and denosumab experienced accelerated fusion rates than with teriparatide alone, which correlated with heightened measures of bone formation [77]. This suggests that the two medications administered together have a heightened effect compared to teriparatide alone.

A recent study by Tani et al. showed that denosumab treatment alone strengthened pedicle screw fixation—with stronger compression force and pullout strength—and increased BMD around the pedicle screw placement [78]. However, these outcomes were not based on analyses after spine surgery but rather on finite element analysis, a computer model generated from measured patient characteristics to simulate vertebral properties [78]. Future investigation with clinical studies analyzing the effects of denosumab therapy on outcomes following spine surgery is required.

#### 4.3.2. Romosozumab

Romosozumab is a monoclonal antibody that binds and inhibits sclerostin, a protein secreted by osteocytes that inhibits osteoblast function and increases RANKL which activates osteoclasts [79]. Thus, romosozumab is unique in that it increases bone formation and decreases bone resorption. Studies in rat models of lumbar fusion had demonstrated increased fusion rates and increased trabecular bone area in animals treated in a dose–response fashion with romosozumab after twice weekly injections for 8 weeks [80]. Mikula et al. found a significant improvement in CT-scan-based Hounsfield units of the lumbar spine by 26% after treatment with romosozumab for a mean length of 10.5 months [81]. When compared with patients treated with teriparatide, denosumab, and alendronate, romosozumab were able to achieve a more substantial improvement in bone density in a shorter duration of time. While many groups have advocated for this use in the spinal deformity population based on anecdotal experience, long-term outcome data are not yet published, but studies are currently ongoing [82,83,84].

### 4.4. Parathyroid Hormone (PTH) Analogs

#### 4.4.1. Teriparatide

PTH analogs regulate the calcium and phosphate metabolism in bone and the kidneys. Counterintuitively, PTH increases bone resorption, thus resulting in increased serum calcium levels. However, low-dose and intermittent exposure (i.e., once daily) disproportionately activate osteoblasts with increased serum calcium more than osteoclast function, thus having a net effect of increased bone mineral density. In a multi-center, prospective randomized study by Ebata et al., 6 months of weekly teriparatide injections significantly increased the rate of bone fusion following posterior or transforaminal lumbar interbody fusion compared to non-treated controls [85]. A retrospective study with patients who underwent posterolateral fusion surgery found that the benefits of teriparatide on bone fusion were significantly greater for periods of treatment longer than 6 months [86]. These results are consistent with systematic reviews and meta-analyses where pooled analysis showed that patients with teriparatide treatment experienced higher fusion rates compared to placebo controls [75,87]. A meta-analysis and retrospective study found that teriparatide-treated patients had decreased subsequent vertebral fractures compared to non-teriparatide-treated patients [87,88].

In a clinical trial, the incidence of pedicle screw loosening was significantly less in patients administered with teriparatide prior to lumbar spinal fusion surgery compared to those with no therapy [76]. Teriparatide administration prior to fusion surgery in osteoporotic postmenopausal patients increased the insertional torque of pedicle screws during surgery, suggesting greater purchase of the screws to the bone [89]. However, conflicting evidence in a recent study shows no significant difference in pedicle screw loosening in teriparatide-treated patients [90]. Additionally, pooled analysis by Fatima et al. found a trend towards a reduced likelihood of pedicle screw loosening in the teriparatide group, but this was not significant [87].

The mechanical benefits of teriparatide after spinal fusion are consistent with improvements in radiographical measures, as evidenced by decreased sagittal misalignment and a mean loss of correction in the local kyphosis angle in patients treated with teriparatide [87,90]. In terms of clinical outcomes, results from meta-analyses show that patients receiving teriparatide following spinal fusion were less likely to experience pain, despite a minimal effect on disability scores, compared to non-teriparatide patients [71,75,87].

Studies have also compared teriparatide to bisphosphonate treatment following spine surgery. A study by Seki et al. found that patients with teriparatide treatment before surgical correction for adult spinal deformity had significantly higher rates of fusion than those treated with bisphosphonates [91]. Similarly, a prospective study by Ohtori et al. showed that the incidence of fusion was larger and the duration until fusion was shorter in patients with teriparatide compared to bisphosphates following posterolateral fusion [92]. These results are consistent with several systematic reviews and meta-analyses showing that teriparatide usage significantly increases the likelihood of fusion compared to bisphosphonates [72,73,87].

In a clinical trial by Ohtori et al., the incidence of pedicle screw loosening was significantly lower in patients administered with teriparatide prior to lumbar spinal fusion surgery compared to those with bisphosphonates [76]. A single-institution study found that by one-year post-surgery, the incidence of pedicle screw loosening was reduced in patients with teriparatide compared to the bisphosphonate group following transforaminal interbody fusion [93]. Regarding clinical outcomes, based on a retrospective study and a prospective clinical trial, patients undergoing spinal fusion did not have differing pain scores depending on if they were given teriparatide or bisphosphonates [92,93].

Together, there is a substantial amount of literature promoting the use of teriparatide in spine surgery to improve the success and strength of fusion. Additionally, there is compelling evidence suggesting a greater mechanical benefit from teriparatide than bisphosphonates in osteoporosis patients undergoing spinal fusion, although there are minimal differences in pain and disability scores (Figure 2D–F). Based on a systematic review, the College of Neurological Surgeons provides an evidence-based guideline recommending teriparatide treatment in osteoporotic patients undergoing spine surgery because it increases BMD, results in earlier and more robust fusion, and improves patient outcomes [94]. Similarly, an expert consensus study among 18 panelists suggests a best practice guideline supporting anabolic agents, including teriparatide, as a first-line treatment for patients with increased risk of fracture undergoing spinal reconstruction, due to their bone-building properties [95].

#### 4.4.2. Abaloparatide

Most studies on parathyroid hormone analogs pertain to teriparatide. However, preliminary evidence suggests that abaloparatide may be beneficial in reducing complications following spine surgery. One study by Arlt et al. found that abaloparatide increased the levels of bone fusion markers in rats that underwent posterolateral fusion [96]. At 28 days post-surgery, 50% of the abaloparatide-treated rats exhibited bilateral fusion compared to only 25% of the controls [96]. While there is limited data evaluating the efficacy of abaloparatide in spine-deformity outcomes, other high-quality studies have demonstrated significant benefit in subsets of patients. Matsumatoto et al. found a statistically significant 12.5% increase in lumbar spine BMD in a randomized, double-blind, placebo-controlled study of postmenopausal women and men with osteoporosis receiving daily injections of 90 micrograms of abaloparatide for 78 weeks [97]. Miller et al. performed a phase 3 double blind RCT in postmenopausal women with osteoporosis and found morphometric vertebral fractures occurred less frequently in the active treatment groups vs. placebo, with greater BMD increases [98]. As this medication has gained more widespread use, ongoing and future studies will more accurately describe the efficacy of this medication in the spinal deformity population.

## 5. Future Research Plans

Optimizing the treatment protocols for adult spinal deformity patients, particularly focusing on the long-term outcomes of newer anabolic agents like abaloparatide and romosozumab is an imperative next step. Additionally, studies could explore the cost-effectiveness and accessibility of these therapies. Research could also investigate the combination of various pharmacological agents with surgical techniques, such as cement augmentation or the use of larger interbody cages, to determine the best strategies for improving surgical outcomes in patients with compromised bone quality. Further studies might aim to assess the effectiveness of preoperative bone health optimization, particularly in high-risk populations, and develop standardized guidelines for bone health management in spinal deformity surgeries.

## 6. Conclusions

Several pharmacological and surgical solutions have been proposed to optimize bone health in patients with spinal deformity requiring nonoperative and operative management. Prevention remains the mainstay, with emphasis on lifestyle, nutrition, weight-bearing activity, and routine age-appropriate screening. In addition to the longstanding efficacy of vitamin and mineral supplements, bisphosphonates, and PTH-analogs, newer anabolic agents and monoclonal antibody treatments show great promise, with ongoing studies to demonstrate their short- and long-term value in reducing complications after spinal deformity surgery. Cost remains a significant hurdle, since despite their known efficacy, access and insurance coverage remains highly variable from patient to patient. Focus on cost-effective strategies will no doubt be a major contributor to the future utilization and outcome measures for these medications.

## Figures and Tables

**Table 1:** Pharmacologic treatment options for bone health optimization.

Class	Agent	Brand Name	Mechanism of Action
Vitamin and mineral supplements	Calcium	N/A	The main mineral component of bone. Forms calcium salts (mostly calcium phosphate) by osteoblasts which harden cartilaginous bone matrices and thus bone building
	Vitamin D	N/A	Activates intestinal absorption of calcium and maintaining calcium homeostasis
Antiresorptive agents	Bisphosphonates	Reclast, Boniva, Fosamax, Zoneta, Actonel Aclasta	Inhibits osteoclast function, thus allowing osteoblasts to more efficiently build bone mass
Anabolic agents	Denosumab	Prolia	Monoclonal antibody that inhibits receptor activator of nuclear factor kappa-B ligand (RANKL), resulting in decreased osteoclast development
	Romosozumab	Evenity	Monoclonal antibody that binds and inhibits sclerostin, a protein secreted by osteocytes that inhibits osteoblast function increases RANKL which activates osteoclasts (PMID: 30775535). Thus, romosozumab is unique in that it increases bone formation and decreases bone resorption
Parathyroid hormone (PTH) analogs	Teriparatide	Forteo	Regulates calcium and phosphate metabolism in bone and the kidneys. Counterintuitively increases bone resorption, thus resulting in increased serum calcium levels. However, low-dose and intermittent exposure (i.e., once daily) disproportionately activate osteoblasts with increased serum calcium more than osteoclast function, thus having a net effect of increased bone mineral density
	Abaloparatide	Tymlos	Similar to teriparatide, but with different pharmacokinetics that may confer some advantages in bone mineral density improvements
